# Initial experience of thoracoscopic segmentectomy of basal segment through the inferior pulmonary ligament approach in treating congenital lung malformations in children

**DOI:** 10.1186/s12887-023-04289-3

**Published:** 2023-09-13

**Authors:** Rui Guo, Jike Liu, Yunpeng Zhai, Huashan Zhao, Hongxiu Xu, Longfei Lv, Shisong Zhang

**Affiliations:** 1 Department of Thoracic and Tumor Surgery, Children’s Hospital Affiliated to Shandong University, Jinan, 250022 Shandong China; 2 Department of Thoracic and Tumor Surgery, Jinan Children’s Hospital, Jinan, 250022 Shandong China; 3 Department of Pediatric Surgery, Liaocheng People’s Hospital, Liaocheng, 252000 Shandong China

**Keywords:** Thoracoscopy, Congenital lung malformation, Segmentectomy, Posterior basal segment

## Abstract

**Purpose:**

This study aimed to evaluate the feasibility and limitations of thoracoscopic segmentectomy of the basal segment (S10).

**Methods:**

Clinical data of 15 children with congenital lung malformations (CLM) who underwent thoracoscopic segmentectomy of S10 via the inferior pulmonary ligament approach from January to October 2022 were retrospectively analyzed. The demographics, clinical presentation, intraoperative time, blood loss, postoperative events, and follow-up duration were assessed.

**Results:**

There were 15 patients in this group (nine males and six females). Age ranges from 4.3 to 96.0 months (median, 7.7 months). Fourteen patients underwent S10 segmentectomy, with one undergoing right S10 segmentectomy and right S6 partial wedge resection. The surgical time was 57–125 min (median, 80 min), intraoperative bleeding volume (5–20 ml; median, 10 ml), postoperative drainage tube indwelling (2–4 d; median, 3 d), and postoperative hospitalization time (4–7 d; median, 5 d). No intraoperative conversions, surgical mortalities, or major complications were observed among these patients. Subcutaneous emphysema appeared in three patients; however, it disappeared following conservative observation without pneumothorax or bronchopleural fistula occurrence.

**Conclusions:**

Thoracoscopic segmentectomy of S10 via the inferior pulmonary ligament approach is technically feasible for treating CLM; however, this surgical approach may have certain limitations for CLM with large cysts.

## Introduction

Congenital lung malformations (CLMs) are characterized by airway dysplasia, lung parenchyma, and pulmonary vessels. Congenital pulmonary airway malformations (CPAM) and intra-lobar pulmonary sequestration (ILS) are the most common types [1]. Current treatment strategies favor early surgery due to the risk of infection and malignant transformation [2]. Further, with advances in minimally invasive technologies, thoracoscopic lung resection has increasingly gained popularity [3]. Thoracoscopic segmentectomy is becoming more common in children with small lesions (limited to one or more lung segments). The posterior basal segment (S10) segmentectomy is particularly challenging in lung segment resection and has seldom been performed in children [4]. All these surgeries in children are performed through the hilar approach, which faces with such an inevitable problem. Because S10 is far from the hilum, exposing the hilum of S10 requires not only necessary breaking the connection between the dorsal (S6) and basal segments but also fully freeing the hilum, which increases the possibility of postoperative lung torsion and extensive damage to the lung tissue, with obvious drawbacks. The thoracoscopic S10 segmentectomy in adult had previously been performed. Based on these issues, some scholars have used the inferior pulmonary ligament approach to perform S10 segmentectomy and achieved satisfactory surgical results [5–8]. We drew on the surgical experience of adults and performed thoracoscopic segmentectomy of S10 through the inferior pulmonary ligament approach in 15 children with CLM from March to November 2022, exploring the feasibility and limitations of this surgical procedure in treating CLM.

## Methods

The Ethics Committee of Jinan Children’s Hospital (Children’s Hospital Affiliated to Shandong University) approved this study, and it followed the principles of the Declaration of Helsinki. The parent of each child provided written informed consent.

### Patients

**Fig. 1:**
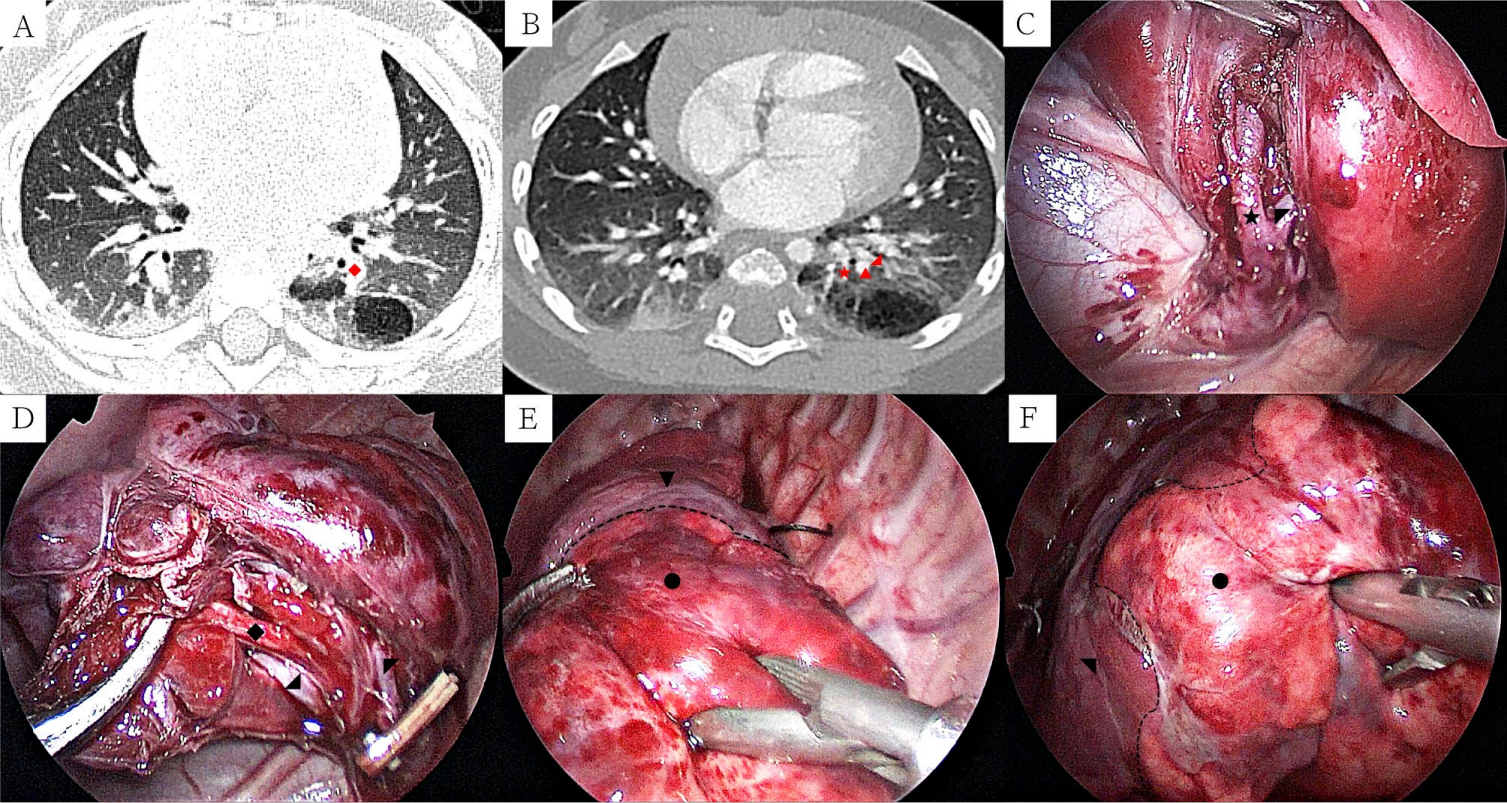
Preoperative CT and key surgical procedures of thoracoscopic segmentectomy of the left S10 via the inferior pulmonary ligament approach: (1 **A**) The lesion is located on the left S10, and B10 enters the lesion (lung window); (1**B**) Important structures that need to be cut off in left S10: B10, A10, V10, and V9 located at the boundaries of the lesion can be preserved (mediastinal window); (1 **C**) Separate the lower pulmonary vein and expose V10 and V6; (1**D**) After cutting off V10, separate and expose B10 from V9 and V6; (1**E**) Modified “expansion and collapse method” to determine the plane between segments, showing the boundary between S6 and S10; (1 F) Modified “expansion and collapse method” to determine the plane between segments, indicating the boundary between S9 and S10. (S6: ▼, S10: ●, S9: ◥, B10: ◆, A10: ▲, V10: ★, V9: ◢, V6: ◤)

**Fig. 2:**
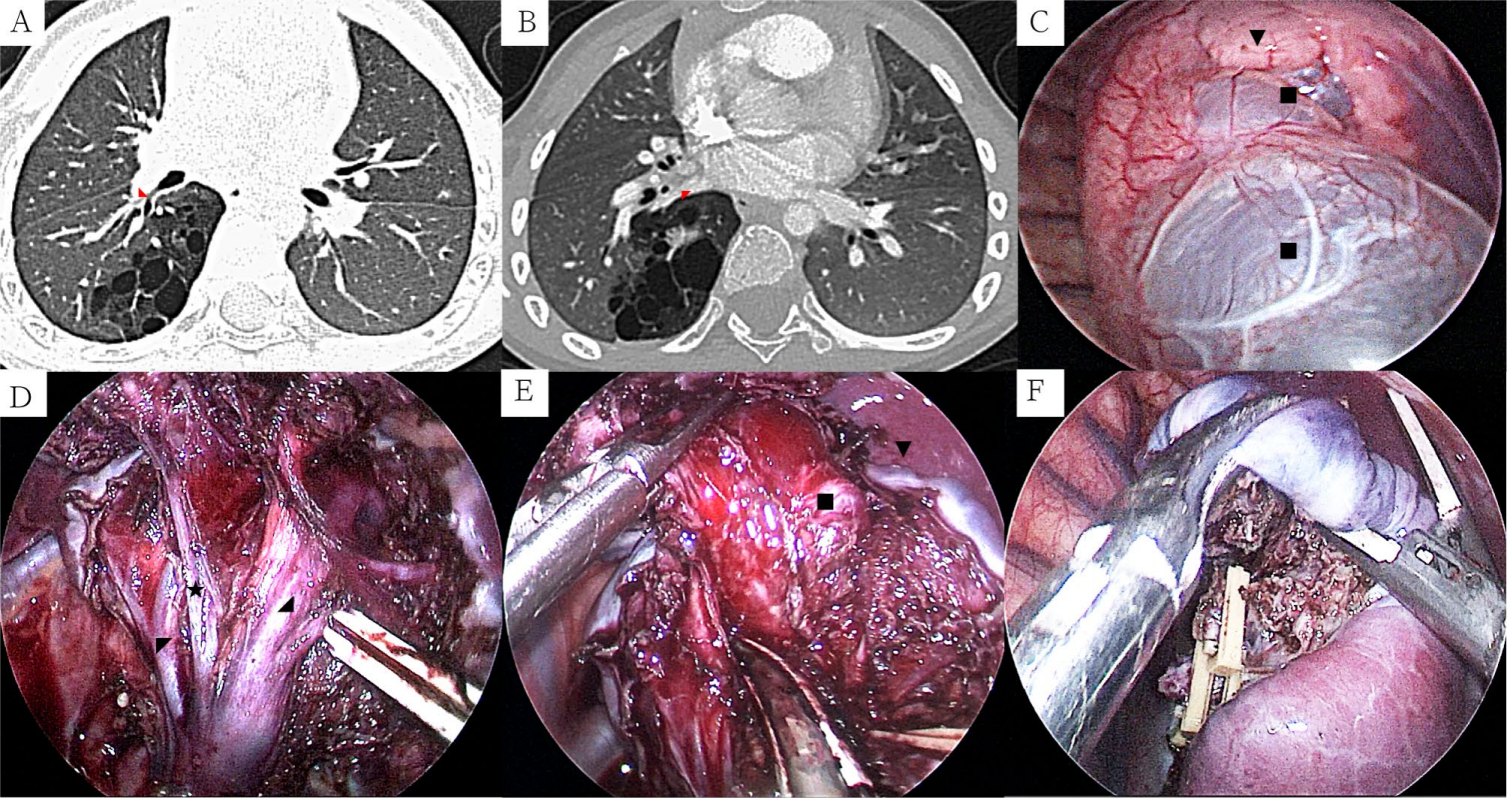
Preoperative CT and key surgical procedures of thoracoscopic segmentectomy of right S10 and partial wedge resection of right S6: (2 **A**) decreased S6 due to lesion compression, B6 did not enter the lesion (lung window); (2**B**) V6 was located at the boundaries of the lesion and had not entered into the lesions (mediastinal window); (2 **C**) Cystic dilation of the lesion, with some cysts penetrating into S6; (2**D**) Determined the inter-segment plane by the freed inter-segment veins (V6, V9); (2**E**) Peeled off cysts deep into S6; (2 F) After successful dissection of deep cysts, some S6 marginal tissue blackened out and was resected. (Large cysts: ■, S6: ▼, V10: ★, V9: ◢, V6: ◤, B6: ◣)

In our hospital, 15 children with CLM underwent thoracoscopic S10 resection through the inferior pulmonary ligament approach between January and October 2022. All preoperative patients underwent enhanced chest CT to confirm the diagnosis, determine the abnormal supply of blood vessels (location, quantity, and diameter), and design a surgical plan(Figs. 1A and B and 2A and B). In addition, basic patient information, CLM characteristics (specific type and lesion location), and peri-operative data (operation time, blood loss, drainage tube use duration, complications, and postoperative hospital stay duration) were recorded for all patients. Patient screening criteria: (1) patients with CPAM or ILS; (2) the lesion is limited to unilateral S10, and there is no acute inflammatory response; (3) the diameter of the lesion should be ≥ 2 cm. Exclusion criteria were as follows: Children with concomitant diseases that affect cardiopulmonary function, such as congenital heart disease, restrictive or obstructive chest wall disease, and other deformities necessitating simultaneous surgery.

### Surgical technique

Single-lumen tracheal intubation (selective closure of the affected main or basal bronchi with bronchial occlusion) was performed to achieve single-lung ventilation. An artificial pneumothorax was established at a pressure of 4–8 mmHg (1 mmHg = 0.133 kPa) and a flow rate of 1–2 L/min. A three-hole method was used to perform surgery in the side-lying position. The observation hole was located at the 7th intercostal line of the subscapular angle. The two operating holes were located at the 8th intercostal line of the axillary midline and the 8th intercostal line between the vertical spinal muscle line and the subscapular angle line. The chest cavity was accessed, and the inferior pulmonary ligament was severed with an electric hook to expose the lower pulmonary vein and totally free the lower pulmonary vein tributaries (V6) and common basal vein (CBV). Next, dissociate V10 along the CBV, detach V10, and completely dissociate V9 from its deep surface. Next, locate and totally separate A10 and B10 between V6 and V9 (Fig. 1C and D). After ligating and clamping A10 and B10, the lungs were fully inflated, and the modified “dilation collapse method” was used to determine the inter-segment plane of S10 (Fig. 1E F). However, suppose the modified “dilation collapse method” cannot be used to determine the inter-segment plane of S10 in children clearly. In that case, the inter-segment veins (V6, V9) can be used (Fig. 2D). LigaSure™ was used to seal the lung tissue along the inter-segment plane of S10 and completely remove S10. The resected lung tissue was removed from the retrieval bag, and the chest was rinsed with warm physiological saline. Sections of each blood vessel, bronchial stump, and lung tissue were carefully examined to ensure no active bleeding or air leakage before suturing with the stamp edge with 5 − 0 prolene. The lung was expanded sufficiently, and a closed thoracic drainage tube was implanted at the 7th intercostal line of the subscapular angle. The chest tube was removed when there was no air leakage, and the amount of daily drainage was < 20mL.

### Statistical analysis

Continuous data were presented as medians and ranges, and categorical variables were presented as frequencies (%). Table 1 shows the clinical parameters. SPSS (Windows version 23.0; IBM Co., Armonk, NY, USA) was used for all statistical analyses.

**Table 1:** Clinical data of children undergoing segmentectomy of S10 through the inferior pulmonary ligament approach

Patient number	Gender	Age (months)	Weight(kg)	Symptomatic (Y/N)	Type of CLM	Type of resection	Operative time(min)	Blood loss(ml)	Drainage duration (days)	Duration of post-operative hospital stay(days)	Complications	Follow-up time(months)
1	F	10.0	9.5	Y	ILS	segmentectomy	106	10	3	5	N	5
2	F	5.0	7.0	N	ILS	segmentectomy	75	15	3	6	N	5
3	F	11.4	9.0	N	CPAM	segmentectomy	81	5	2	4	N	6
4	M	18.0	12.5	N	ILS	segmentectomy	65	10	2	4	N	6
5	M	6.3	8.5	N	CPAM	segmentectomy	57	20	3	5	N	7
6	M	4.5	8.0	Y	ILS	segmentectomy	109	10	3	5	N	8
7	F	7.7	10.0	N	CPAM	segmentectomy	80	10	3	5	N	9
8	M	96.0	27.0	Y	ILS	segmentectomy	61	5	3	6	N	9
9	M	58.0	17.0	Y	CPAM	segmentectomy and partial wedge resection	125	10	4	6	N	9
10	M	7.5	9.5	N	CPAM	segmentectomy	57	10	2	4	N	10
11	F	17.0	9.5	Y	ILS	segmentectomy	88	20	3	6	subcutaneous emphysema	10
12	F	4.6	8.0	N	CPAM	segmentectomy	58	5	4	7	subcutaneous emphysema	12
13	M	6.3	9.5	N	ILS	segmentectomy	85	10	2	4	N	13
14	M	31.0	16.5	Y	CPAM	segmentectomy	103	15	3	5	N	13
15	M	4.3	7.0	N	CPAM	segmentectomy	61	5	3	6	subcutaneous emphysema	13

## Results

**Fig. 3:**
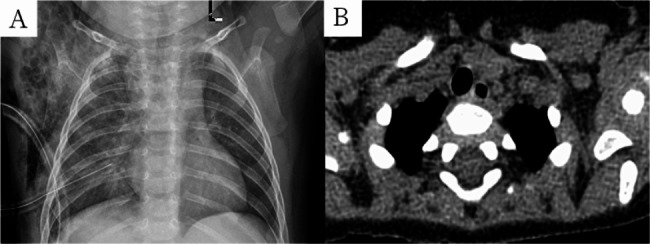
Recovery status of children with subcutaneous emphysema after surgery: (3 **A**) Chest X-ray showing subcutaneous emphysema on the right side 3 d after surgery; (3**B**) One month after surgery, chest CT showing the disappearance of subcutaneous emphysema on the right side

There were 15 patients in this group (nine males and six females). Age ranges from 4.3 to 96.0 months (median, 7.7 months). Weight ranged from 7.0 to 27.0 kg (median, 9.5 kg). Thirteen cases of CLM were diagnosed using prenatal ultrasound and chest CT examination within 3 months after birth, including seven cases of CPAM and six cases of ILS. Owing to recurrent respiratory tract infections, two patients were diagnosed using chest CT, including one case of CPAM and one case of ILS. Lesions in six cases were located on the right S10, whereas those on the left S10 were found in nine. The lesion diameters of the lesions range from 2.8 to 5.9 cm (median, 4.3 cm). Six children underwent surgery following rehabilitation for recurring clinical respiratory symptoms (cough in four cases and wheezing in two). Nine were asymptomatic, but their parents requested active surgical treatment (Table 1). The surgery was uneventful for all 15 patients. The surgical time was from 57 to 125 min, with a median of 80 min, whereas the intra-operative bleeding ranged from 5 to 20 ml, with a median of 10 ml. In this study, nine patients underwent segmentectomy of the left S10, and five underwent segmentectomy of the right S10. One child was scheduled to undergo a segmentectomy of the right S10. Furthermore, when the lesion was completely excised, the surrounding tissue of S6 was very thin, and blood flow was inadequate due to large cysts in the lesion compressing the adjacent lung segment. Therefore, segmentectomy of the right S10 and partial wedge resection of the right S6 were performed (Fig. 2C F). The postoperative drainage tube indwelling lasted 2–4 d, with a median of 3 d, whereas the postoperative hospitalization time lasted 4–7 d, with a median of 5 d. These patients had no intraoperative conversions, surgical mortalities, or major complications. Three patients were diagnosed with subcutaneous emphysema. After conservative observation, the subcutaneous emphysema resolved, and no pneumothorax or bronchopleural fistula occurred (Fig. 3). Table 1 summarizes the operative and postoperative outcomes.

Follow-up visits and chest CT were performed 3 months, 6 months, and 1 year after surgery. All 15 patients were followed-up on without being missed. During the follow-up period (median, 9 months; range, 5–13 months), no patients presented with hemoptysis, and no residual lesions were observed on chest CT. In addition, symptoms either completely resolved or significantly improved in all symptomatic patients, and no complications were detected throughout the follow-up period.

## Discussion

The incidence of CLM is 1–4.2 per 10,000 [9]. The CLM primarily consists of CPAM and PS. For children with CLM having no obvious symptoms after birth, there is some controversy over whether to choose conservative observation or surgical resection [10]. Due to the risk of infection and malignant transformation associated with asymptomatic children, surgical treatment has been able to achieve low risk and low complications in managing CLM. Therefore, it is recommended to choose surgical treatment for asymptomatic CLM children [11].

Owing to the fact that CLM is a benign lesion, segmentectomy is a good choice for patients whose lesions are limited to the lung segment if the technical level is mature. Lung segments were divided into simple or complex segments. Simple segmental resection, similar to lobectomy, is a relatively simple and widely performed surgery. Complex lung segment resection is challenging because of the need to manage multiple segment planes. S10 Surgery is more challenging since it is far from the hilum of the lungs and adjacent to three or four lung segments [12, 13]. Segmentectomy through the hilar approach is currently the most commonly used method for S10 resection. This method can be used to achieve S10 segmentectomy; its drawbacks are undeniable. To expose the hilar of S10, the lower pulmonary artery, lower pulmonary bronchus, and their tributaries should be extensively freed, which is very traumatic. Moreover, the connection between S6 and the basal segment should be severed. After removal, S10, S6, and S7 (8) -9 are in a free state, increasing the risk of pulmonary torsion. Owing to the fact that the end of the pulmonary vein is close to the hilum of the lung, accurately distinguishing the inter-segment veins from the intra-segment veins is difficult while freeing the pulmonary vein from the end to the main trunk. It is easy to rupture inter-segment veins inadvertently, obstructing blood flow return [14]. Based on the characteristics of S10, some researchers have proposed an inferior pulmonary ligament approach for S10 resection. The deep surface of the inferior pulmonary ligament directly corresponds to the S10 segment gate, which can be rapidly released. The probability of lung torsion is reduced by maintaining the link between S6 and S7 (8) -9. The dissociation of branches from the main trunk of the lower pulmonary vein can easily expose intra-segmental and inter-segmental veins, which is beneficial for preserving the inter-segmental veins. Good surgical outcomes have been achieved (5–8). S10 segmentectomy is now rarely performed in children, and all procedures are performed through the hilar approach [4, 15]. Therefore, for lesions in S10, we perform segmentectomy via the inferior pulmonary ligament approach to explore the feasibility and limitations of this surgery for treating CLM in children.

Children have a smaller thoracic space than adults do, and an artificial pneumothorax is more likely to affect hemodynamics significantly. Therefore, establishing a suitable operating space is critical to a successful surgery. By combining an artificial pneumothorax with selective single-lung ventilation, we established a surgical operating space. During surgery, we found that the inferior pulmonary ligament approach made it easier to construct an operating area. Only one-lung ventilation in the lower lobe is required to obtain sufficient operating space because there is no need to consider the impact of the upper and middle lobes on the operating space after establishing an artificial pneumothorax and completing diaphragm compression. This significantly reduces the technical requirements for anesthesiologists and makes surgery easier. It is necessary to break the cysts to gain a larger space for patients with CLMs and large cysts [16]. CLM differs from lung cancer. This is because it is a benign cystic lesion requiring no surgical margin consideration. This only necessitates complete resection of the lesions. Is there a more suitable method for determining the inter-segment plane? The modified “dilation and collapse method,” whose purpose is to find the target segment bronchus is currently the most often used method for establishing the inter-segment plane [17, 18]. Owing to the fact that the target segment bronchus is relatively fixed in position, it can be found between V6 and V9 at the V10 deep surface after disconnecting V10 [5, 8]. Owing to the young age of children, the target bronchus is very small, the wall is very thin, and dissociation requires extra caution and time. The modified “dilation and collapse method” cannot be used to determine the inter-segment plane once the target segment bronchus ruptures. It is necessary to destroy the large cysts to obtain a wider operating space for children with CLM accompanied by large cysts. In this situation, using the modified “dilation and collapse method” to determine the inter-segment plane often yields poor results. In response to the limitations of the modified “dilation collapse method” in determining the inter-segment plane of the CLM children, we often combine the use of inter-segment veins for inter-segment plane determination. Intersegmental veins are natural boundaries between lung segments. The inter-segmental plane can be determined if accurately positioned and dissociated [19]. The tissues of children are loose, blood vessels are more prone to dissociation, and they bleed less, making them easier to operate. Preoperative 3D reconstruction is useful for surgical planning; however, it might be challenging to achieve good outcomes in children due to their small blood vessels. Therefore, 2D CT readings can be used to clearly define the venous route and plan surgery, requiring surgeons to have a good ability to read CT films [20]. Moreover, for surgeons with advanced CT film-reading ability, determining and dissociating the segmental veins (intra-segmental-and inter-segmental veins) is easy, and segmentectomy can be accomplished by the inter-segmental vein defining the inter-segment plane. In this group cases, we used the modified “dilation and collapse method,” supplemented by the inter-segmental vein to determine the inter-segmental plane, and successfully completed 14 cases of S10 resection. Cystic lesions are common in CLM, and the characteristics of cystic dilation can affect segmentectomy. In this study, we found that the deep surface of the lesion located in S10 penetrated the adjacent lung segment, pressing the deep tissue of the adjacent lung segment outward in children with CLM accompanied by large cysts. Further, when the lesion is entirely excised, the surrounding tissue of the adjacent lung segment is very thin and has a poor blood supply. Except for S10 segmentectomy, a partial wedge resection of the adjacent lung segment is often required. One child in this group was scheduled to undergo a segmentectomy of the right S10. The segmentectomy of the right S10 and partial wedge resection of the right S6 were performed due to the large cyst in the lesion penetrating the next lung segment. Air leakage is a common complication following segmentectomy, with severe cases leading to bronchopleural fistula [21, 22]. Only a few patients in this group experienced minor air leakage and subcutaneous emphysema, which quickly resolved after surgery. This is because surgery through the inferior pulmonary ligament does not involve the problem of managing a large airway. Hence, even if air leakage occurs when the target bronchus is properly treated, only the alveolar air leakage can self-close. We sutured the stump edge to reduce the risk of air leakage further.

Our center has achieved good short-term clinical outcomes in treating CLM using thoracoscopic segmentectomy of S10 via the inferior pulmonary ligament approach. However, the sample size of this study was small, and it was a single-center, retrospective study. Therefore, it will be necessary in later stages to further verify the feasibility and limitations of this surgical method in treating patients with CLM using large-sample, prospective, and multicenter studies. In this study, we found that there are challenges in determining the inter-segment plane and performing precise segmentectomy of S10 using only the modified “dilation and collapse method” for CLM with large cysts. Therefore, other surgical methods are more suitable for these patients. Some researchers have recently proposed surgical methods, such as “modified wedge resection” and “anatomical lesion resection.” Theoretically, the core of these surgical methods is to perform precise resection of the lesion via inter-segmental veins as the internal boundary and the natural boundary between the lesion and normal tissue as the external boundary, rather than being limited to precise segmentectomy [23, 24]. Therefore, it is worth exploring whether this surgical method is more advantageous in treating CLM with large cysts.

## Conclusion

Thoracoscopic segmentectomy of S10 via the inferior pulmonary ligament approach is technically feasible for treating patients with CLM; however, this surgical method may have certain limitations for CLM with large cysts.

## Data Availability

The data used to support the findings of this study are included in the article.
